# Predicting Risks of Machine Translations of Public Health Resources by Developing Interpretable Machine Learning Classifiers

**DOI:** 10.3390/ijerph18168789

**Published:** 2021-08-20

**Authors:** Wenxiu Xie, Meng Ji, Riliu Huang, Tianyong Hao, Chi-Yin Chow

**Affiliations:** 1 Department of Computer Science, City University of Hong Kong, Kowloon, Hong Kong 518057, China; Vasiliky@outlook.com (W.X.); chiychow@cityu.edu.hk (C.-Y.C.); 2 School of Languages and Cultures, University of Sydney, Sydney 2006, Australia; rhua5035@uni.sydney.edu.au; 3 School of Computer Science, South China Normal University, Guangzhou 510631, China; haoty@m.scnu.edu.cn

**Keywords:** multinominal naïve bayes classifier, public health education and promotion, machine learning, digital vulnerability

## Abstract

We aimed to develop machine learning classifiers as a risk-prevention mechanism to help medical professionals with little or no knowledge of the patient’s languages in order to predict the likelihood of clinically significant mistakes or incomprehensible MT outputs based on the features of English source information as input to the MT systems. A MNB classifier was developed to provide intuitive probabilistic predictions of erroneous health translation outputs based on the computational modelling of a small number of optimised features of the original English source texts. The best performing multinominal Naïve Bayes classifier (MNB) using a small number of optimised features (8) achieved statistically higher AUC (M = 0.760, SD = 0.03) than the classifier using high-dimension natural features (135) (M = 0.631, SD = 0.006, *p* < 0.0001, SE = 0.004) and the automatically optimised classifier (22) (M = 0.7231, SD = 0.0084, *p* < 0.0001, SE = 0.004). Furthermore, MNB (8) had statistically higher sensitivity (M = 0.885, SD = 0.100) compared with the full-feature classifier (135) (M = 0.577, SD = 0.155, *p* < 0.0001, SE = 0.005) and the automatically optimised classifier (22) (M = 0.731, SD = 0.139, *p* < 0.0001, SE = 0.0023). Finally, MNB (8) reached statistically higher specificity (M = 0.667, SD = 0.138) compared to the full-feature classifier (135) (M = 0.567, SD = 0.139, *p* = 0.0002, SE = 0.026) and the automatically optimised classifier (22) (M = 0.633, SD = 0.141, *p* = 0.0133, SE = 0.026).

## 1. Introduction

Public health discourse is a heterogeneous system which encompasses clinical, research, and educational resources. Clinical resources are known for their lexical, syntactic irregularities introduced by clinical professionals to medical records [1,2]. Research resources are known for their linguistic, content complexity, formality [3,4,5]. Both genres pose significant challenges for machine translation (MT) technologies which are susceptible to language difficulty and irregularity [6,7,8]. Compared to these specialised discourses, public health educational resources developed by health authorities are designed to have high information accessibility, as these resources are intended to inform, guide, and support the general public in acquiring an essential understanding of health risks and diseases. Given the important social function of public health education resources, the translation of original English health resources at national and international health authorities is often made available through professional translation for not only English-speaking populations but also people and communities from linguistically and culturally diverse backgrounds. For many years, the World Health Organization (WHO) has been a champion in multilingual health communication and promotion. Public health educational resources developed by health authorities such as the WHO have significantly less lexical, syntactic irregularities compared to clinical writings and controlled language difficulty compared to research or policy materials.

With increasing cost-effectiveness and practical convenience, machine translation is having increasing applications in online health education and promotion. Neural MTs (NMTs) such as Google Translate have significantly improved their performance over traditional statistical MT tools, especially the linguistic fluency and acceptability of MT outputs [9,10,11,12]. MT tools are not only used extensively by professional translators but also by global multilingual users, especially those with limited English proficiency and health literacy, in search for authoritative health information in order to facilitate the understanding and acquisition of latest reliable health knowledge. Our study aimed to explore the risks of MT tools in the translation of online health promotion resources for non-English speaking readers. We developed risk-prevention mechanisms, i.e., machine learning classifiers for risk prediction, to enable a safer and more informed use of MT tools in health promotion and to close the gap in the provision of quality health information to vulnerable populations and communities.

## 2. Related Work

Machine translation (MT) is having increasing applications in healthcare settings to facilitate the communication and interaction between medical professionals and multicultural and multilingual populations including migrants, ethnic minorities, and refugees. A growing number of studies have shown that despite the convenience, low-cost, and increasing accuracy of MT tools, there are still considerable risks in the use of current translation technologies in healthcare settings. The development of effective risk-prevention mechanisms will enable a safer and more informed use of MT tools and help close the gap in technology-assisted healthcare to provide better quality health services to vulnerable populations and communities. Much of current studies on the evaluation of MT output are based on lexical, grammatical, or syntactic properties of the language. The authors of [13] used Naive Bayes, decision tree, and support vector machine classifiers in order to automatically detect statistical machine translation errors in Brazilian Portuguese, including number agreement, gender agreement, verb inflectional errors, part of speech errors, extra word, missing word, untranslated word, incorrectly translated word, misspelling, missing, untranslated or mistranslated multiword expressions, and reordering words. The decision tree algorithm correctly classified 76.7% of the instances using the 5-W (a token window of size five), whereas Naive Bayes and SVM achieved their best overall precision using the 7-W (73.6 and 74.5%, respectively). However, such studies rarely explored erroneous MT output in the context of clinical and health research by using machine learning methods. In our study, we detected and classified some important types of MT errors which are clinically significant, especially for vulnerable users of MT tools who have very limited bilingual skills and medical knowledge, as well as medical professionals who might use MT tools to communicate with their patients.

## 3. Materials and Methods

### 3.1. Collection and Selection of WHO Public Health Education Resources

We systematically searched for articles on public health education on infectious diseases from the website of the WHO (May 2021) under the section of Health Topics (www.who.int/health-topics/, accessed on 31 May 2021). We reviewed and retained 185 articles for subsequent corpus annotation and statistical analyses. The standard structure of articles in the Health Topics section included an overview of the disease, its symptoms, and treatment. Articles that appeared in sections other than Health Topics such as WHO news/media, research publications, or policy materials were not included in the data collection to control for the content difficulty and the target readerships of the original English health materials to be used for developing machine learning algorithms. The finalized sample covered more than 30 global and regional leading infectious diseases: human immunodeficiency virus, Dengue fever, Ebola virus, Lassa fever, Yellow fever, Zika virus, foodborne botulism, tetanus, campylobacteriosis, Brucellosis, Cholera, Shiga toxin-producing Escherichia coli, waterborne Legionella pneumophila, plague, Salmonella, Salmonella Typhi, mycotoxins, herpes simplex virus type 1 and type 2, lymphatic filariasis, dracunculiasis, Schistosomiasis, human monkeypox, leprosy, malaria, human papillomavirus, cervical cancer, Middle East respiratory syndrome coronavirus, listeriosis, leishmaniasis, and so on.

### 3.2. Statistical Analysis

Flesch Reading Ease [14], Gunning Fog Index [15], and SMOG Index [16] were used to assess the linguistic difficulty of the original English health resources. The results revealed a similarly high level of language difficulty of the WHO public health materials: Flesch Reading Ease (M = 32.805, SD = 12.706, SE = 0.934, 95% CI: 30.974, 34.636); Gunning Fog Index (M = 14.441, SD = 2.634, SD = 2.634, SE = 0.194, 95% CI: 14.062, 14.821); SMOG Index (M = 14.822, SD = 2.016, SE = 0.148, 95% CI: 14.532, 15.113). For Flesch Reading Ease, a mean score of 32 indicates the suitability of these English health resources for senior college students; for Gunning Fog and SMOG Index, a mean score higher than 14 also suggests that these materials were accessible only by highly educated readers. Existing research shows that linguistic difficulty caused by syntactic and lexical complexity can cause an impact on the quality of automatic translation outputs. However, the applicability of readability tools as input features for the development of machine learning algorithms remains understudied.

## 4. Screening and Classification of Machine Translation Mistakes

In order to develop machine learning classifiers to assess the risk profile or suitability of original English health education and promotion materials for machine translation, we first identified the human translations of the original English texts collected. The WHO has 5 official languages, and the English resources we collected on the WHO website have been mostly translated to the other 4 official languages including the target language under study, Chinese. Human translations were matched pairwise with machine translation outputs by Google Translate of the same English materials. Two bilingual translators compared and assessed differences between paired human and machine translations. Linguistic differences between the paired translations that did not cause barriers of understanding of the Chinese translations or any clinically significant misunderstanding of the original English materials were allowed as acceptable translation variations. Machine translation divergences from their matching human translations, which was perceived concurrently by both bilingual translators (Cohen’s kappa coefficient 0.941, Asymptotic Standard Error = 0.026, 95% CI: 0.89, 0.992) as clinically significant, were highlighted. The review of machine translation outputs identified 4 large categories of automatic translation issues of the WHO standard public health education promotion resources: conceptual mistakes, mistranslation of disease symptoms, incomprehensible translations, and ambiguous translations causing difficulties in understanding the translations. The first two types of translation errors (Examples 1–9) are clinically significant, as without the correction of the erroneous machine translation outputs by bilingual medical professionals, the translated information can cause misdiagnosis of diseases or even result in life-threatening behaviours. The last two types of automatic translation issues (Examples 10–11) were related to the suboptimal translation outputs of low understandability and low translated health information usability.

## 5. Classification of Clinically Significant MT Errors

In our study, to demonstrate the significant impact of English health resources on MT (Google Translate) output quality, we selected standard health information on infectious diseases from the World Health Organization (WHO) as training and testing data for developing MCLs using multinomial Naive Bayes (MNB). Mistakes in MT Chinese outputs exhibited variability in terms of accuracy of clinical information (epidemiology, symptoms, and diagnosis) and communicative effectiveness (logical confusion).

Diverse machine translation issues identified through double-blind human evaluation revealed an important fact that the original English materials associated with erroneous or suboptimal and less effective MT outputs did not involve any complex medical terminologies or jargon as conventionally believed (Table 1). Instead, most of these translation mistakes occurred with high frequency and low difficulty vocabulary (see Appendix A) such as ‘onset’ (example 1), ‘domestic’ and ‘semi-domestic’ (example 2), ‘drug solutions’ (example 3), ‘event driven’ (example 4), ‘culling’ (example 5), ‘outbreak’ (example 6), ‘delivery’ (example 7), ‘occasionally’ (example 9). It was suspected that the occurrence of such inconspicuous yet clinically significant medical and health translation mistakes was due to the limited ability of current neural MT systems to correctly interpret and discriminate the meaning of polysemous words between general versus specialised texts such as medical and health materials.

The meaning of polysemous words that are predominant in general textual materials tends to be prioritized in machine translation outputs. In health and medical domain, this translation bias can cause clinically significant, misleading, or incomprehensible translation outputs. These translation issues, however, are less conspicuous or detectable than earlier MT systems such as statistical MT, as neural machine translation outputs are characterized by their improved fluency and idiomaticity, as noted by Way in the following [17].

[Neural] MT output can be deceptively fluent; sometimes perfect target-language sentences are output, and less thorough translators and proofreaders may be seduced into accepting such translations, despite the fact that such translations may not be an actual translation of the source sentence at hand at all!

For end users of these MT tools, a lack of adequate medical training or health literacy can significantly increase the risks of using MT tools to acquire critical health knowledge and public health advice from health authorities such as the WHO.

## 6. Training and Testing of Machine Learning Classifiers

We divided the total sample set into 70% training (129) and 30% testing (56) data. We manually classified the original English materials as MT-error-prone English texts (EPET) and non-MT-error prone English texts (NEPET) in order to develop supervised machine learning classifiers for predicting the likelihood of MT errors (falling under any of the MT mistakes defined in this study, such as conceptual mistakes, symptom mistakes, and incomprehensible or ambiguous/confusing translation outputs), given the features of the original English health materials. In the training data, the ratio of EPET and NEPET was 1.35 (EPET: 74; NEPET: 55). In the testing data, the ratio of EPET and NEPET was 0.87 (EPET: 26; NEPET: 30).

Various English corpus annotation systems exist. In our study, we chose to annotate the WHO original English public health materials by using Readability Studio (Oleander Software Ltd., Vandalia, OH, USA) and USAS (University of Lancaster Semantic Annotation System) [18] to annotate the original English health materials from the WHO website. The use of Readability Studio was for verifying the hypothesis that the language difficulty of the original English texts was the main factor contributing to the occurrence of clinically significant errors in machine translation outputs. There is a rich literature on the impact of source texts on the quality of translations. Readability Studio generated descriptive statistics of a total of 20 morphological, structural, and lexical features of the original English texts including average number of sentences per paragraph; number of difficult sentences (more than 22 words); longest sentence; average sentence length; number of unique words; number of syllables; average number of characters; average number of syllables; number of proper nouns; number of monosyllabic words; number of unique monosyllabic words; number of complex (more than three syllable) words; number of unique (more than there) syllable words; number of long (more than six characters) words; number of unique long words; misspellings; overused words; wordy items; passive voice; and sentences that begin with conjunctions.

The use of the USAS semantic annotation system was based on our observation of the patterns of clinically significant errors in machine translation outputs as shown in the illustrative examples. It was high frequency polysemous words that tended to cause mistakes in automatic translations, instead of morphological or syntactically complex expressions. Semantic annotation will help explore the relations between the semantic meanings of original English expressions and the errors that occurred in the machine translation results. The USAS semantic system annotated the original English health texts with 115 finely classified semantic features (general or abstract terms A1–A15, body/the individual B1–B5, arts/crafts C1, emotion E1–E6, food/farming F1–F4, government/public G1–G3, housing/home H1–H5, commerce/industry I1–I4, sports/games K1–K6, living creatures L1–L3, movement/location/transport M1–M8, and numbers/measurements N1–N6; substances/materials/objects/equipment O1–O4, education P1, language/communication Q1–Q4, social actions, states, and processes S1–S9; time T1–T4; world and environment W1–W5, psychological actions, states, processes X1–X9, science and technology Y1–Y2, and names and grammar Z1–Z99). The use of USAS was based on the observation of the importance of the discrimination of semantic meanings by neural machine translation tools such as Google Translate to produce reliable and accurate health translation outputs.

## 7. Optimisation Techniques

The total number of features of the annotated English health materials was 135. Small (185) and high-dimensional (135) data such as ours can add uncertainty or cause overfitting to machine learning classifiers being developed. In order to increase the performance of machine learning algorithms, we used a backward selection method and recursive feature elimination (RFE) with support vector machine (SVM) as the base estimator to optimize the original high-dimensional feature set. We applied two optimisation techniques to identify the best feature set using minimal classification error (MCE) as the selection criterion. Machine learning is distinct from statistical modelling in that the relevance of a certain feature largely depends on the other features included in the machine learning model. Features which have statistically significant differences in two samples might not be considered by machine learning as top discriminating features. It is the interaction of features which affects the performance of machine learning classifiers. The two optimisation techniques we used were based on two different hypotheses: hypothesis 1 states that errors in machine translations of public health resources were due to morphological, structural and lexical complexity and difficulty of the resources, and hypothesis 2 states that errors in machine translations of public health resources were due to the combined morphological, structural, lexical, and semantic complexity of these resources, particularly the technically challenging issue of automatic translation of polysemous words in specialised heath domains. This was based on our observation of the original WHO resources in English and their machine translations to Chinese. Subsequently, we applied two optimisation techniques to test these two hypotheses. First, we applied separate optimisation on the marked morphological, structural, or lexical (MSL) features (20 in total) and the annotated semantic features (115). This resulted in two separate optimised feature sets: the optimised MSL feature set (5) which contained the number of difficult sentences, average number of characters, average number of syllables, passive voice, and sentences beginning with conjunctions; and the following optimised semantic feature set (10): A14 (exclusivizers and particularizes), A7 (probability), A8 (seem), H5 (furniture, household fittings), I1 (money generally), L1 (life and living things), N6 (frequency), S3 (relationship), S5 (groups, affiliation), and X9 (ability). Next, we applied joint optimisation of two combined feature sets to explore the interaction between morphological, structural, lexical, and semantic features using the same backward elimination method. An optimised feature set through joint optimisation (22) emerged which included 2 syntactic and 20 semantic features: number of difficult sentences, average number of characters, A7 (probability), A14 (exclusivizers, particularizes), B1 (anatomy, physiology), B5 (clothes, personal belongings), E2 (liking), F3 (smoking, non-medical drugs), F4 (farming, horticulture), K1 (entertainment), L2 (living creatures), M8 (stationary), O4 (physical attributes), Q4 (media), S1 (social actions, states, processes), S3 (relationship), S7 (power relationship), W5 (green issues), X3 (sensory), X7 (wanting; planning; choosing), X8 (trying), and X9 (ability).

## 8. Refinement of Automatically Optimised Features

In order to develop interpretable machine learning classifiers to predict the likelihood of machine translation errors, we reviewed the jointly optimised features (CFJO 22) and further reduced the number of features in this automatically selected feature set on the basis of the interpretability of semantic features. Only features which were linguistically relevant in health and medical materials were retained: B1 (anatomy, physiology), L2 (living creatures), S1 (social actions, states, processes), and X9 (ability). In order to compensate for the loss of model accuracy of the jointly optimised feature after manual feature elimination and refinement (refine CFJO_6), we added two semantic features based on observation of the patterns of errors in automatic translation outputs: A13 (degree) and T2 (time). We also compared the relative performance of binary classifiers using the five optimised MLS features to further improve the performance of the refined jointly optimised feature set. Table 2 shows the area under the receiver operating characteristic curves (ROC) of the binary classifiers using the independently optimised morphological, lexical, and syntactic features: number of difficult sentences, average number of characters, average number of syllables, passive voice, and sentences that begin with conjunctions. It shows that the number of difficult sentences (22 words or more) (AUC = 0.534, 95% CI: 0.45, 0.62) and passive voice (AUC = 0.532, 95% CI: 0.45, 062) had the two highest AUCs. 

**Figure 1:**
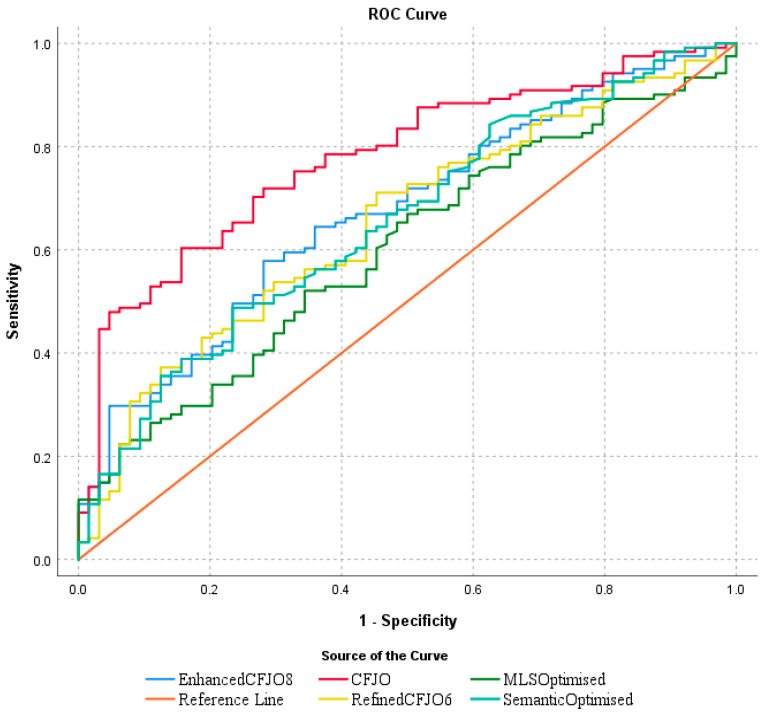
AUC of binary classifiers using different optimised feature sets.

Figure 1 and Table 3 show the AUCs of binary logistic regressions with different optimised feature sets as independent variables in order to predict the probabilities of the original WHO English health materials as machine translation error prone texts. It shows that among the five optimised feature sets, the optimised feature set through joint optimisation achieved the highest AUC (0.778), followed by the enhanced joint optimisation which included five features from the automatic joint optimisation and three features added manually following a close examination of the linguistic relevance of automatically selected semantic features (semantic optimisation 10) and a comparison of the relative performance of automatically selected morphological, lexical, and syntactic features (MLS optimisation 5) (Table 3).

## 9. Multinominal Naïve Bayes (MNB) Classifiers

Multinomial Naïve Bayes (MNB) classifier is a Bayes theorem-based statistical classification algorithm and is shown to be effective for categorical text data analysis [19]. Based on Bayes theorem, MNB is effective, robust, and highly scalable, which works well on small data and can develop simple and powerful models for disease prediction [20,21]. Moreover, the MNB classifier performs probabilistic learning that computes the posterior probability of the given sample which can be applied for further decision making [22,23]. In this research, the MSL and semantic features are represented as the number of feature occurrences in the original text, i.e., bag-of-words features, which makes MNB more suitable than other Naïve Bayes classifiers such as Bernoulli Naive Bayes (assumes that the features are binary) and Gaussian Naive Bayes (assumes that the features are continuous and follow the normal distribution). Furthermore, the object of our research is to learn how likely an English source text would prone a machine translation error. Thus, the Naïve Bayes classifier (MNB) is more suitable than other machine learning models in our study for ensuring interpretability and reliability of the learned classifiers. We performed 5-fold cross-validation (on the training data) and hold-out validation (trained on training data and evaluated on testing data) in order to assess the performance of MNB under different settings.

## 10. Results

### 10.1. Comparison of Performance of Classifiers

Table 4 shows the results of the training and testing of MNB classifiers with different feature sets. It shows that machine learning classifiers outperformed binary classifiers using scores of popular readability tools as predicting features that had very low specificity. Comparing the performance of MNB classifiers using full feature sets and separately optimised feature sets, it was found that the sole optimisation of morphological, lexical and syntactic (MLS) features from 20 to 5 improved the model sensitivity from 0.4615 (SD = 0.1565) to 0.9615 (SD = 0.0604) but decreased specificity from 0.7 (SD = 0.1339) to 0.0677 (SD = 0.0555); the sole optimisation of semantic features from 115 to 10 decreased sensitivity from 0.8846 (SD = 0.1003) to 0.7692 (SD = 0.1322) and increased specificity from 0.4333 (SD = 0.1448) to 0.5667 (SD = 0.1448) and AUC from 0.6538 (SD = 0.0475) to 0.7256 (SD = 0.0145). Automatic joint optimisation of the combined MLS and semantic features (CFJO_22) achieved higher specificity (M = 0.6333, SD = 0.1408) than separately optimised and combined MLS and semantic features (CFSO) (M = 0.500, SD = 0.1460) but achieved lower sensitivity (M = 0.7308, SD = 0.1392) than CFSO (M = 0.7692, SD = 0.1322). Lastly, MNB with manually refined and enhanced feature sets (CFJO_6 and CFJO_8) through the adjustment of CFJO (22) achieved the highest sensitivity (CFJO_6: M = 0.840, SD = 0.1132; CFJO_8: M = 0.8846, SD = 0.1003), specificity (CFJO_6: M = 0.633, SD = 0.1408; CFJO_8: M = 0.6667, SD = 0.1377); AUC (CFJO_6: M = 0.759, SD = 0.0797; CFJO_8: M = 0.7603, SD = 0.0301), and accuracy (CFJO_6: M = 0.7397, SD = 0.1270; CFJO_8: M = 0.7679, SD = 0.1190).

### 10.2. Area under the Curve of Receiver Operator (AUC of ROC), Sensitivity, and Specificity

**Figure 2:**
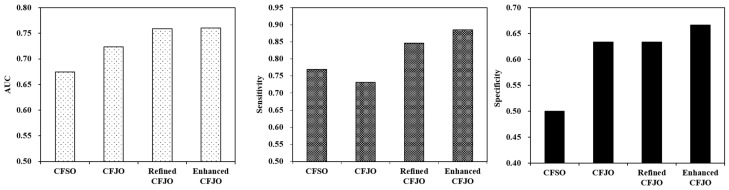
Evaluation of MNB classifier with different optimised features on the test data.

Figure 2 and Table 5 display the statistical differences between MNB classifiers using four different optimised feature sets: separately optimised and then combined MLS and semantic features (CFSO) (15), jointly combined features (CFJO) (22), and two optimised CFJO feature sets that include refined CFJO (6) and enhanced CFJO (8). In terms of AUC, CFJO (22) was statistically better than CFSO (*p* < 0.0001, 95% CI of AUC mean difference: 0.0403–0.0571, dCohen = 2.177, 95% Confidence Interval for dCohen: 1.516–2.838); enhanced CFJO (8) was statistically improved than the automatically constructed CFJO (22) (*p* < 0.0001, 95% CI of AUC mean difference: 0.0746–0.0972, dCohen = 2.835, 95% Confidence Interval for dCohen: 2.093–3.577). The refined CFJO (6) was also statistically improved than automatically constructed CFJO (22) (*p* < 0.0001, 95% CI of AUC mean difference: 0.0620–0.1072, dCohen = 1.402, 95% Confidence Interval for dCohen: 0.817–1.987); however, the difference between refined CFJO (6) and enhanced CFJO (8) was not statistically significant (*p* = 0.9093, 95% CI of AUC mean difference: −0.0213–0.0239, dCohen = 0.022, 95% Confidence Interval for dCohen: −0.502–0.545).

In terms of sensitivity, the enhanced CFJO (8) was statistically improved than both automatically constructed CFJO (22) (*p* < 0.0001, 95% CI of AUC mean difference: 0.1084–0.1992, dCohen = 1.268, 95% Confidence Interval for dCohen: 0.694–1.842) and automatically constructed CFSO (15) (*p* < 0.0001, 95% CI of AUC mean difference: 0.0715–0.1593, dCohen = 0.983, 95% Confidence Interval for dCohen: 0.429–1.538). The refined CFJO (6) was statistically improved than the automatically constructed CFJO (22) (*p* < 0.0001, 95% CI of AUC mean difference: 0.0617–0.1567, dCohen = 0.861, 95% Confidence Interval for dCohen: 0.313–1.408); refined CFJO (6) was also statistically improved than enhanced CFJO (8) (*p* = 0.0294, 95% CI of AUC mean difference: 0.0045 to 0.0847, dCohen = 0.417, 95% Confidence Interval for dCohen: −0.112–0.947).

In terms of specificity, CFJO (22) was statistically better than CFSO (*p* < 0.0001, 95% CI of AUC mean difference: 0.0796 to 0.1870, dCohen = 0.929, 95% Confidence Interval for dCohen: 0.3781.481); the enhanced CFJO (8) was statistically improved than the automatically constructed CFSO (*p* < 0.0001, 95% CI of AUC mean difference: 0.1136 to 0.2198, dCohen = 1.175, 95% Confidence Interval for dCohen: 0.607 to 1.742). The refined CFJO (6) was statistically improved than the automatically constructed CFSO (*p* < 0.0001, 95% CI of AUC mean difference: 0.0793–0.1867, dCohen = 0.927, 95% Confidence Interval for dCohen: 0.376–1.479); however, the difference between refined CFJO (6) and enhanced CFJO (8) was not statistically significant (*p* = 0.2071, 95% CI of AUC mean difference: −0.0856–0.0188, dCohen = −0.242, 95% Confidence Interval for dCohen: −0.768–0.284).

### 10.3. Classifier Scalability

**Figure 3:**
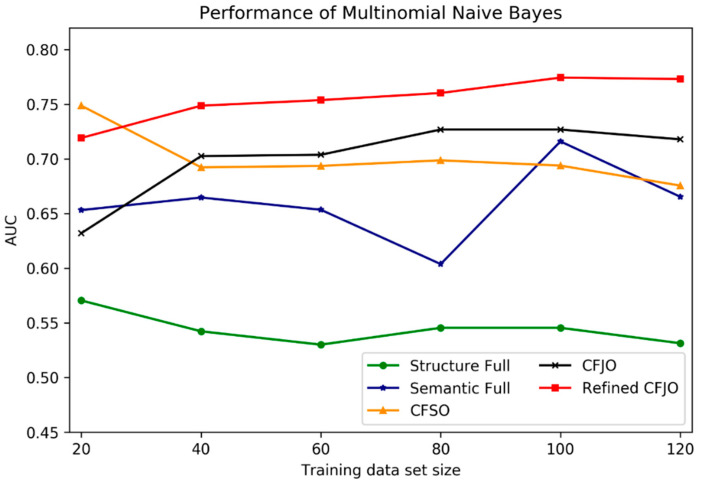
AUC of MNB on testing data with different feature set and different training dataset size.

In order to evaluate robustness and scalability of the MNB classifiers, we evaluated the performance of MNB on different sizes of the training data (with 20, 40, 60, 80, 100, and 120 samples) when using different features sets. Furthermore, the Mann–Whitney U test was applied to evaluate the statistical significance of the differences in the AUCs in different settings (using different features and different training data set sizes). The performance was validated on the testing data (56 samples) in terms of AUCs. The comparison results were shown in Figure 3 and Table 6. Regardless of the changes in the training data sizes, MNBs with MSL full (AUC Mean: 0.5442) and enhanced CFJO (AUC Mean: 0.7549) features were more robust and stable than other feature sets, with the lowest standard deviations of 0.0146 and 0.0203, respectively. The performances of MNBs with Semantic full (AUC Mean: 0.6594) and CFJO (AUC Mean: 0.7017) features were more fluctuated when the size of training data changed, with the largest standard deviations of 0.0359 and 0.0357, respectively. MNB with the enhanced CJFO features achieved the highest AUC mean 0.7549 when comparing to the classifiers using other feature sets.

In terms of the validation of scalability of MNBs, we performed the Mann–Whitney U test to access the statistical significance of different features with the training dataset sizes changed. The enhanced CFJO was statistically improved compared to the other four features sets, which the *p*-values of paired Mann–Whitney U test were all less than 0.05. CFSO was statistically better than both MSL and semantic full features, with *p*-values of 0.005 (95% CI of AUC mean difference: 0.1316 to 0.1809) and 0.045 (95% CI of AUC mean difference: −0.0508 to 0.1329), respectively. CFJO was also statistically better than both MSL full features with a *p*-value of 0.0049 (95% CI of AUC mean difference: 0.0377 to 0.1928). The statistical results demonstrated that the MNB with the enhanced CFJO feature had better stability, scalability, and robustness, which was less likely to be affected by the training data size and worked well on small data.

## 11. Discussions

### Probabilistic Outputs

Table 7 shows outputs of the readability tool based binary classifiers and MNB classifiers as probabilities of belonging to either machine translation error-prone and non-error-prone English public health materials. Readability tools-based classifiers and outputs of MNB classifiers used two full feature sets: MLS (20) and semantics (115) and their separately optimised feature sets MLS optimised (5) and semantics optimised (10) did not differ significantly between English health materials prone to machine translation errors and those which were not prone to machine translation errors. The outputs of MNB classifiers using separately optimised and then combined feature sets (CFSO) and jointly optimised feature set (CFJO) and the derived refined CFJO models (refined CFJO; enhanced CFJO) differed significantly between two sets of English materials on infectious diseases. 

**Figure 4:**
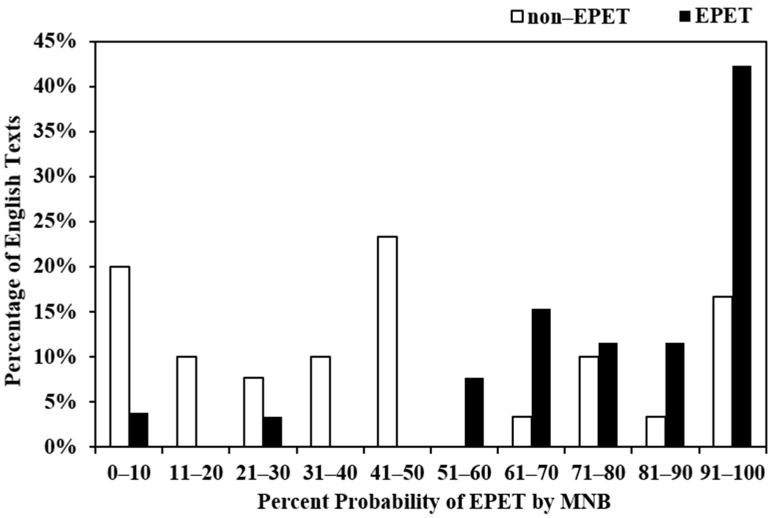
Percentage of non-MT error prone and MT-error-prone English health materials assigned by MNB classifier to each 10% probability bin.

Figure 4 is a histogram that shows the number of non-MT error prone (non-EPET) and MT-error-prone English (EPET) health materials that fell into each 10% probability bin based on MNB output. Sixty-seven percent of non-MT error prone English health materials were assigned a probability of risk-free texts <50% (specificity = 0.67), and 88% of MT-error-prone English health materials were assigned a probability of risky texts ≥50% (sensitivity = 0.88).

In Figure 4, about 12% of MT-error-prone WHO English public health educational and promotion materials were assigned low probabilities of 0–10% and 21–30%. In order to improve the classifier sensitivity and prediction accuracy, one method is to adjust probability thresholds in order to achieve the desired sensitivity and specificity pairing. Table 8 shows that if we decrease the current classifier probability threshold from 0.5 to 0.2, the model sensitivity increases from 0.8846 (95% CI: 0.762, 1.00) to 0.9615 (0.888, 1.0), but specificity decreases from 0.667 (95% CI: 0.498, 0.835) to 0.33 (95% CI: 0.165, 0.502). By contrast, if we increase the probability threshold from 0.5 to 0.8 while specificity increases to 0.667 (95% CI: 0.498, 0.835) to 0.80 (95% CI: 0.657, 0.943), the sensitivity decreases from 0.8846 to 0.5385 (95% CI: 0.347, 0.730). The diagnostic utility (positive likelihood ratio LR+, negative likelihood ratio LR−) is another effective measurement of the effectiveness of the assessment tool. Positive likelihood ratio is the ratio between sensitivity and false positivity (1-specificity), and negative likelihood ratio is the ratio between false negativity (1-sensitivity) and specificity. Large positive likelihood ratios and small negative likelihood ratios are indicators of good assessment tools. Table 8 shows that 0.5 is the best probability threshold for the best performing MNB classifier using the enhanced CFJO (8) feature set which includes two MLS features of the number of difficult sentences and passive voice and six semantic features of B1 (anatomy, physiology), L2 (living creatures), S1 (social actions, states, processes), X9 (ability), A13 (degree), and T2 (time).

## 12. Conclusions

The main purpose of our study was twofold: first, to develop machine learning classifiers as decision aids and help vulnerable non-English speaking people to appreciate the risks of using MT tools to seek and acquire health information online. Second, to develop machine learning classifiers as a risk-prevention mechanism to help medical professionals with little or no knowledge of the patient’s languages in order to predict the likelihood of clinically significant mistakes or incomprehensible MT outputs based on the features of English source information as input to the MT systems. An important contribution of our study was the development of interpretable machine learning models to help assess and predict the risks of using machine learning tools to translate public health educational and promotion resources developed by international health organization such as WHO. The application of the machine learning classifier in clinical or health research settings requires an understanding of its functionality.

The linguistic cues that the MNB classifier uses to predict whether a certain English health text is likely to be translated erroneously by Google Translate are the syntactic structure and the semantic meanings of neighboring words in the vicinity of the actual translation mistake. We call these neighboring words as indicative contextual features (ICF). The best performing classifier we developed succeeded in classifying MT-error-prone and non-MT-error prone English texts (185) using eight ICFs, with sensitivity of 88%, specificity of 67%, and AUC of 0.76. It should be noted that the occurrence of translation errors does not require the presence of all eight ICFs in an English text. The illustrative examples show that the patterns of the distribution of these ICFs are diverse, which could be an MLS feature (number of difficult sentences) and one or a few semantic ICFs or a combination of semantic ICFs or an MLS feature alone. The empirical evidence of our study has challenged the traditional view that mistakes or suboptimal translation outputs were largely due to the presence of complex medical jargons as hallmarks of medical discourse.

Our study revealed that with rapid development in computer science, the accuracy of machine translation such as neural machine translation tools is improving significantly. The translation of medical terminology and jargons no longer represents the top challenge in automatic translation technologies. Rather, it is linguistic phenomena such as polysemy or context-dependent nature of common words in specialised health and medical domains that are causing subtle yet clinically significant errors and confusion in machine translation outputs. The development of machine learning classifiers could help add protection to MT-assisted health education to vulnerable communities and people by allowing health and medical professionals to have a reliable, real-time estimate of the risks of using MT tools to engage with people with limited English and health literacy.

Promoting the safe and risk-aware use of MT technologies to enhance access to public health resources and services represents an understudied field of health communication. Despite widely reported issues and risks of MT tools, there is a persistent lack of research tools to help reduce and minimize the risks of using MT mediated health communication among multilingual and multicultural populations. We developed machine learning classifiers to provide intuitive probabilistic prediction of the risks of translating certain English health resources using Google Translate online to translate to the Chinese language, which can be conveniently adapted for other languages in order to optimize and increase the safety of cross-lingual online health information dissemination.

## Figures and Tables

**Table 1:** Types of clinically misleading machine translation errors.

Types	Subtypes	Examples (For Full Examples See Appendix A) (Translated to Chinese)
Conceptual mistakes (word level)	Related to epidemiology such as disease or virus transmission	English: longer *onset* times for lower levels of intoxication. Human: it took longer for symptoms to reveal when the intoxication was low. MT: the attacks of symptoms took longer when the intoxication was low.
		English: different mosquito species live in different habitats; some breed around houses (*domestic*), others in jungles (wild), and some in both habitats (*semi-domestic*). Human: different mosquito species live in different habitats; some breed around houses (domestic), others in jungles, and some in both environments (semi-domestic). MT: different mosquito species live in different habitats; some breed around houses (national), others in jungles, and some in both places (semi-national).
	Related to medical, clinical, and procedural measures	English: serological or other testing; *culling* can be effective in areas with low prevalence. Human: serological or other testing; killing can be effective in areas with low prevalence. MT: serological or other testing; removal can be effective in areas with low prevalence.
Logical Confusion (sentence level)	Related to epidemiology such as disease or virus transmission	English: *occasionally*, humans working or travelling in the forest are bitten by infected mosquitoes and develop yellow fever. Human: People working or travelling in the forest are occasionally bitten by infected mosquitoes and develop yellow fever. MT: People who occasionally work or travel in the forest are bitten by infected mosquitoes and develop yellow fever.
	Related to medical, clinical, and procedural measures	English: medical male circumcision reduces the risk of heterosexually acquired HIV infection in men by approximately 50%, including in ‘real world’ settings where scale up occurred *alongside the increasing coverage of ART with its secondary prevention effect*. Human: Medical male circumcision can reduce the risks of men contracting HIV through heterosexual activities by about 50%, including in ‘real world’. With the increasing application of antiretroviral therapy and its secondary prevention effect, this method (medical male circumcision) was on the rise. MT: Medical male circumcision can reduce the risks of men contracting HIV through heterosexual activities by about 50%, including in ‘real world’. With the increasing application of ART, its secondary prevention effect increased as well.
		English: a person wanting to know his HIV status collects a specimen, performs a test, and interprets the test results *in private or with someone they trust*. Human: a person wanting to know his HIV status can privately or with someone they trust collect a specimen, perform a test, and interpret the test results. MT: a person wanting to know his HIV status can collect a specimen, perform a test, and then privately or explain the test results to people they trust.

Note: Underlined text: This is the where the translation mistake occurs.

**Table 2:** Area Under the ROC Curve of independently optimised morphological, lexical, and syntactic features.

Test Result Variable	AUC	S.E.	Asymptotic Sig ^a^	Asymptotic 95% CI	
Number of difficult sentences	0.534	0.043	0.428	0.450	0.618
Average number of characters	0.474	0.045	0.566	0.386	0.563
Average number of syllables	0.500	0.045	0.992	0.411	0.588
Passive voice	0.532	0.044	0.461	0.447	0.618
Sentences that begin with conjunctions	0.508	0.006	0.156	0.497	0.520

^a^ Null hypothesis: true area = 0.5.

**Table 3:** Area Under the ROC Curve of binary classifiers using different optimised feature sets.

Optimised Feature Sets	AUC	S.E.	Asymptotic Sig ^a^	Asymptotic 95% Confidence Interval	
MLS Optimisation (5)	0.601	0.043	0.018	0.518	0.685
Refined CFJO (6)	0.652	0.042	0.000	0.570	0.733
Semantic Optimisation (10)	0.653	0.042	0.000	0.571	0.735
Enhanced CFJO (8)	0.672	0.040	0.000	0.593	0.752
CFJO (22)	0.778	0.034	0.000	0.711	0.846

^a^ Null hypothesis: true area = 0.5.

**Table 4:** Performance of MNB with Different Feature Sets on Test and Test Datasets.

Optimisation Techniques	Training Data	Test Data			
	AUC Mean (SD)	AUC (SD)	Accuracy (SD)	Sensitivity (SD)	Specificity (SD)
Binary Classifiers using popular readability tools					
Flesch Reading Ease Scores (60)	/	0.3699	0.5000	1.000	0.0667
Gunning Fog Index (12)	/	0.4263	0.5000	0.8462	0.2000
SMOG Index (12)	/	0.4051	0.4643	0.9231	0.0667
MNB with Full, Non-Optimised Feature Sets					
MLS Full Feature Set (20)	0.5638 (0.139)	0.5314 (0.0648)	0.5893 (0.1452)	0.4615 (0.1565)	0.7000 (0.1339)
Semantics Full Feature Set (115)	0.5709 (0.116)	0.6538 (0.0475)	0.6429 (0.1225)	0.8846 (0.1003)	0.4333 (0.1448)
Structure + Semantics Full (135)	0.5776 (0.187)	0.6308 (0.0062)	0.5714 (0.1499)	0.5769 (0.1551)	0.5667 (0.1448)
MNB with Automatically Optimised Feature Sets					
MLS Optimised Feature Set (5)	0.5385 (0.110)	0.5256 (0.0289)	0.4821 (0.0677)	0.9615 (0.0604)	0.0667 (0.0555)
Semantics Optimised Feature Set (10)	0.5529 (0.042)	0.7256 (0.0145)	0.6607 (0.1385)	0.7692 (0.1322)	0.5667 (0.1448)
Combined separately optimised feature (CFSO) (15) (Semantics Optimised 10 + MLS Optimised 5 features)	0.569 (0.068)	0.6744 (0.0305)	0.6250 (0.1392)	0.7692 (0.1322)	0.5000 (0.1460)
MLS-Semantics jointly optimised (CFJO) (22)	0.7194 (0.088)	0.7231 (0.0084)	0.6821 (0.140)	0.7308 (0.1392)	0.6333 (0.1408)
MNB with Refined Optimised Feature Sets with Enhanced Interpretability, Parsimony, Accuracy					
Refine CFJO (6)	0.6561 (0.0927)	0.759 (0.0797)	0.7397 (0.1270)	0.84 (0.1132)	0.633 (0.1408)
Enhanced CFJO (8)	0.6487 (0.080)	0.7603 (0.0301)	0.7679 (0.1190)	0.8846 (0.1003)	0.6667 (0.1377)

**Table 5:** Paired sample t-tests of area under the curve, sensitivity, specificity, effect sizes dCohen, Glass’ Delta Δ, and CLES.

**AUC Classifier** **(Feature Numbers): i, j**	***p***	**SE**	**95% CI of Mean** **Difference (j–i)**	**Effect Size d_Cohen_**	**Effect Size Glass’ Δ**	**Common Language** **Effect Size (CLES)**	**95%** **Confidence Interval for d_Cohen_**
CFSO (15) vs. CFJO (22)	<0.0001	0.004	0.0403–0.0571	2.177	5.798	0.938	1.516–2.838
CFSO (15) vs. CFJO (8)	<0.0001	0.006	0.0746–0.0972	2.835	2.854	0.977	2.093–3.577
CFSO (15) vs. CFJO (6)	<0.0001	0.011	0.0620–0.1072	1.402	1.061	0.839	0.817–1.987
CFJO (22) vs. CFJO (8)	<0.0001	0.004	0.0289–0.0455	1.683	1.236	0.883	1.074–2.293
CFJO (22) vs. CFJO (6)	=0.0011	0.011	0.0147–0.0571	0.634	0.45	0.673	0.097–1.17
CFJO (6) vs. CFJO (8)	=0.9093	0.011	−0.0213–0.0239	0.022	0.043	0.506	−0.502–0.545
**Sensitivity** **Classifier (Feature Numbers)**	***p***	**SE**	**95% CI of Mean** **Difference**	**Effect Size d_Cohen_**	**Effect Size Glass’ Δ**	**Common Language** **Effect Size (CLES)**	**Confidence Interval for d_Cohen_**
CFSO (15) vs. CFJO (22)	0.1373	0.026	−0.0892–0.0124	−0.283	−0.276	0.579	−0.809–0.244
CFSO (15) vs. CFJO (8)	<0.0001	0.022	0.0715–0.1593	0.983	1.151	0.757	0.429–1.538
CFSO (15) vs. CFJO (6)	=0.0029	0.023	0.0247–0.1169	0.575	0.625	0.658	0.041–1.11
CFJO (22) vs. CFJO (8)	<0.0001	0.023	0.1084–0.1992	1.268	1.533	0.815	0.694–1.842
CFJO (22) vs. CFJO (6)	<0.0001	0.024	0.0617–0.1567	0.861	0.965	0.729	0.313–1.408
CFJO (8) vs. CFJO (6)	=0.0294	0.020	0.0045–0.0847	0.417	0.445	0.616	−0.112–0.947
**Specificity** **Classifier (Feature Numbers)**	***p***	**SE**	**95% CI of Mean** **Difference**	**Effect Size d_Cohen_**	**Effect Size Glass’ Δ**	**Common Language** **Effect Size (CLES)**	**Confidence Interval for d_Cohen_**
CFSO (15) vs. CFJO (22)	<0.0001	0.027	0.0796–0.1870	0.929	0.947	0.744	0.378–1.481
CFSO (15) vs. CFJO (8)	<0.0001	0.027	0.1136–0.2198	1.175	1.211	0.797	0.607–1.742
CFSO (15) vs. CFJO (6)	<0.0001	0.027	0.0793–0.1867	0.92	0.945	0.744	0.376–1.479
CFJO (22) vs. CFJO (8)	=0.2071	0.026	−0.0188–0.0856	0.24	0.243	0.567	−0.286–0.766
CFJO (22) vs. CFJO (6)	=1	0.027	−0.0527–0.0527	−0.002	−0.002	0.501	−0.526–0.522
CFJO (8) vs. CFJO (6)	=0.2071	0.026	−0.0856–0.0188	−0.242	−0.239	0.568	−0.768–0.284

**Table 6:** Mann–Whitney U tests for different training sizes and feature sets using the MNB model (bold values are significant).

Test Result Pair(s)	Mean Difference	Asymptotic 95% Confidence Interval		
		Lower	Upper	*p*-Value *
Enhanced CFJO vs. MSL Full	0.2107	0.1466	0.2748	0.004998
Enhanced CFJO vs. Semantic Full	0.09548	0.0261	0.1649	0.005075
Enhanced CFJO vs. CFSO	0.05445	−0.0317	0.1406	0.010272
Enhanced CFJO vs. CFJO	0.05321	0.0177	0.0887	0.012907
CFSO vs. MSL Full	0.15625	0.1316	0.1809	0.004998
CFSO vs. Semantic Full	0.04103	−0.0508	0.1329	0.045328
CFSO vs. CFJO	−0.00123	−0.1171	0.1146	0.297107
CFJO vs. MSL Full	0.15748	0.0637	0.2513	0.004922
CFJO vs. Semantic Full	0.042267	−0.0526	0.1372	0.092125
Semantic Full vs. MSL Full	0.11522	0.0377	0.1928	0.004998

* Mann-Whitney U test (2-tailed).

**Table 7:** Comparison of Readability Formula and MNB Output between Error-Prone and Non-Error-Prone English Texts.

Techniques	MT-Error-Prone Texts	Non-MT Error-Prone Texts	*p* *
	Mean Probability, SD (*n* = 30)	Mean Probability, SD (*n* = 26)	
Flesch Reading Ease Scores (60)	30.9667 (14.51)	34.8462 (11.29)	0.097
Gunning Fog Index (12)	14.3423 (2.34)	14.7067 (3.087)	0.349
SMOG Index (12)	14.8154 (1.88)	15.2133 (2.12)	0.227
MLS Full (20 features)	0.4437 (0.45)	0.3411 (0.413)	0.693
Semantics Full (115 features)	0.8423 (0.32)	0.5645 (0.44)	0.050
MLS + Semantics Full (135)	0.6078 (0.46)	0.4441 (0.47)	0.095
MLS optimised (5)	0.5928 (0.08)	0.579 (0.075)	0.749
Semantics optimised (10)	0.6362 (0.18)	0.5086 (0.17)	0.004
MLS + Semantics separate optimised (CFSO) (15)	0.6378 (0.18)	0.5257 (0.18)	0.026
MLS + Semantics jointly optimised (CFJO) (22)	0.7186 (0.29)	0.4487 (0.36)	0.004
Refined CFJO (6)	0.7520 (0.28)	0.4483 (0.33)	0.001
Enhanced CFJO (8)	0.7543 (0.27)	0.4506 (0.33)	0.001

* means statistical significance.

**Table 8:** Sensitivities, specificities, and positive likelihood ratios with probability thresholds of the MNB with Refined CSJO.

Probability Thresholds	Sensitivity (95% CI)	Specificity (95% CI)	Positive Likelihood Ratio (LR+) (95% CI)	Negative Likelihood Ratio (LR−) (95% CI)
0.2	0.9615 (0.888, 1.00)	0.3333 (0.165, 0.502)	1.4423 (1.1072, 1.8789)	0.1154 (0.0158, 0.8419)
0.4	0.8846 (0.762, 1.00)	0.4333 (0.256, 0.611)	1.5619 (1.1086, 2.1984)	0.2663 (0.0851, 0.8328)
0.5	0.8846 (0.762, 1.00)	0.6667 (0.498, 0.8354)	2.6539 (1.570, 4.4851)	0.17308 (0.058, 0.517)
0.6	0.8077 (0.656, 0.959)	0.6667 (0.498, 0.8354)	2.4231 (1.4125, 4.1568)	0.2885 (0.126, 0.656)
0.8	0.5385 (0.347, 0.730)	0.80 (0.657, 0.943)	2.69 (1.210, 5.988)	0.577 (0.367, 0.907)

## Data Availability

Data available upon request.

## Appendix A. Types of Clinically Misleading Machine Translation Errors

### Appendix A.1. Conceptual Mistakes

**Example 1**
**Original English:** Following inhalation of the toxin, symptoms become visible between 1–3 days, with longer ***onset*** times for lower levels of intoxication.
**Human:** 吸入毒素后，症状在1–3天后可见。中毒程度越低，**症状显露**所需时间越长。
**Human:** After inhaling the toxin, symptoms become visible within 1–3 days. The lower the intoxication, the longer it took for the symptoms to show.
**MT:** 吸入毒素后，症状会在1–3天内出现，中毒程度较低时，**发作**时间较长。
**MT:** After inhaling the toxin, symptoms become visible within 1–3 days. The lower the intoxication, the longer the outbreak.

**Example 2**
**Original:** The different mosquito species live in different habitats—some breed around houses (***domestic****)*, others in the jungle (wild), and some in both habitats (***semi-domestic***).
**Human:** 不同的蚊种生活在不同的栖息地—有些在房屋周围（**家居环境**）繁殖，有些在野外丛林中繁殖，还有些可在两种环境中都繁殖（**半家居环境**）。
**Human:** Different mosquito species live in different habitats—some breed around houses (domestic), others in jungles, some in both environments (semi-domestic).
**MT:** 不同种类的蚊子生活在不同的栖息地—有些在房屋周围繁殖（**国内**），有些在丛林中（野生），有些在两个栖息地（**半国内**)。
**MT:** Different mosquito species live in different habitats—some breed around houses (national), others in jungles and some in both places (semi-national).

**Example 3**
**Original:** People who inject drugs can take precautions against becoming infected with HIV by using sterile injecting equipment for each injection, and not sharing drug-using equipment and ***drug solutions***.
**Human:** 注射吸毒者可以采取措施避免感染艾滋病毒，包括每次注射都使用无菌注射设备，不共享吸毒工具和**毒品溶液**。
**Human:** People who inject drugs can avoid being infected with HIV with preventative measures, such as using sterile injection equipment each time, and not sharing drug equipment and drug solutions.
**MT:** 注射吸毒者可以通过每次注射使用无菌注射设备，并且不共用吸毒设备和**药物溶液**来预防感染艾滋病毒。
**MT:** People who inject drugs can avoid being infected with HIV with preventative measures, such as using sterile injection equipment each time, and not sharing drug equipment and medicine solutions.

**Example 4**
**Original:** For men who have sex with men **“*event driven*” *PrEP*** is also an effective PrEP option. This is taking two pills sex between two and 24 h in before sex: then, a third pill 24 h after the first two pills, and a fourth pill 48 h after the first two pills.
**Human:** 对于男性行为者来说，**“****按需启动****”****暴露前预防**也是一种有效选项。具体就是在发生性行为前2到24小时服用两片；然后,在首次服药后24小时服用第三片，首次服药后48小时服用第四片。
**Human:** For men who have sex with men, prevention before exposure based on ‘driven by needs’ model be an effective option. Specifically, this means to take 2 pills 2–24 h before sex; then, take the 3rd pill 24 h after the first pill, and the fourth pill 48 h after the 4th pill.
**MT:** 对于与男性发生性关系的男性,**“****事件驱动”PrEP**也是一种有效的PrEP选择。这是在性交前的2到24小时之间服用两粒药；然后，在前两粒药丸后24小时服用第三粒药丸，在前两粒药丸后48小时服用第四粒药丸。
**MT:** For men who have sex with men, driven by events PrEP is an effective PrEP option. This is to take 2 pills 2–24 h before sex; then, the 3rd pill 24 h after the first 2 pills and the 4th pill 48 h after the first 2 pills.

**Example 5**
**Original:** Serological or other testing and ***culling*** can also be effective in areas with low prevalence.
**Human:** 血清学或其他检测和**扑杀措施**在低流行率地区也是有效的。
**Human:** Serological or other testing and killing can be effective in areas with low prevalence.
**MT:** 血清学或其他检测和**剔除**在流行率低的地区也很有效。
**MT:** Serological or other testing and removal can be very effective in areas with low prevalence.

**Example 6**
**Original:** HSV-1 (Herpes Simplex) is most contagious during an ***outbreak*** of symptomatic oral herpes but can also be transmitted when no symptoms are felt or visible.
**Human:** 1型单纯疱疹病毒在有症状的口腔疱疹**发作**期间的传染性最强，但当没有症状或者症状不明显时也可出现传播。
**Human:** HSV-1 is most contagious during the outbreak of symptomatic oral herpes but can also be transmitted when no symptoms are shown or less visible.
**MT:** HSV-1在有症状的口腔疱疹**爆发**期间最具传染性，但也可以在没有感觉或可见症状时传播。
**MT:** HSV-1 is most contagious during the outbreak (among people) of symptomatic oral herpes but can also be transmitted when no symptoms are shown or less visible.

**Example 7**
**Original:** The transmission of HIV from an HIV-positive mother to her child during pregnancy, labour, ***delivery*** or breastfeeding is called vertical or mother-to-child transmission (MTCT).
**Human:** 艾滋病毒阳性母亲在妊娠，生产，**娩出**或哺乳期间将艾滋病毒传给婴儿称为垂直传播或母婴传播。
**Human:** The transmission of HIV from an HIV-positive mother to her child during pregnancy, labour, delivery or breastfeeding is called vertical or mother-to-child transmission (MTCT).
**MT:** 艾滋病毒阳性母亲在怀孕、分娩、**分娩**或哺乳期间将艾滋病毒传播给她的孩子称为垂直传播或母婴传播 (MTCT)。
**MT:** The transmission of HIV from an HIV-positive mother to her child during pregnancy, labour, **labour** or breastfeeding is called vertical or mother-to-child transmission (MTCT).

### Appendix A.2. Logical Confusion

**Example 8**
**Original:** HIV self-testing is a process whereby a person who wants to know his or her HIV status collects a specimen, performs a test, and interprets the test results ***in private or with someone they trust.***
**Human:** 当人们希望得知其艾滋病毒感染状况时可以采用自检程序，他们可以私下或与信得过的人一起采集标本，进行检测并解读检测结果。
**Human:** (a person wanting to know his HIV status) can privately or with someone they trust collect a specimen, perform a test, and interpret the test results.
**MT:** HIV自我检测是一个过程，希望了解自己的HIV状态的人收集样本，进行检测，并私下或与他们信任的人解释检测结果。
**MT:** (a person wanting to know his HIV status) can collect a specimen, perform a test, and then privately or explain the test results to people they trust.

**Example 9**
**Original:*****Occasionally*** humans working or travelling in the forest are bitten by infected mosquitoes and develop yellow fever.
**Human:** 在森林中工作或旅行的人**偶尔**会被受感染的蚊子叮咬并染上黄热病。
**Human:** People working or travelling in the forest, occasionally are bitten by infected mosquitoes and develop yellow fever.
**MT:****偶尔**在森林中工作或旅行的人会被受感染的蚊子叮咬并患上黄热病。
**MT:** People who occasionally work or travel in the forest are bitten by infected mosquitoes and develop yellow fever.

**Example 10**
**Original:** Medical male circumcision reduces the risk of heterosexually acquired HIV infection in men by approximately 50% including in ‘real world’ settings where ***scale up occurred alongside the increasing coverage of ART with its secondary prevention effect.***
**Human:** 男性自愿医疗包皮环切术可将男性通过异性性行为方式获得艾滋病毒感染的风险降低50%，包括在“现实世界”环境中。**随着抗逆转录病毒疗法及其二级预防效果覆盖面的扩大，这种做法也在增加**。
**Human:** Medical male circumcision can reduce the risks of men contracting HIV through heterosexual activities by about 50%, including in ‘real world’. With the increasing application of antiretroviral therapy and its secondary prevention effect, this method (medical male circumcision) was on the rise.
**MT:** 医学男性包皮环切术可将男性异性性感染HIV感染的风险降低约50%，包括在“现实世界”环境中，**随着****ART****的覆盖范围不断扩大，其二级预防效果不断扩大**。
**MT:** Medical male circumcision can reduce the risks of men contracting HIV through heterosexual activities by about 50%, including in ‘real world’. With the increasing application of ART, its secondary prevention effect increased as well.
